# ODAE: Ontology-based systematic representation and analysis of drug adverse events and its usage in study of adverse events given different patient age and disease conditions

**DOI:** 10.1186/s12859-019-2729-1

**Published:** 2019-05-01

**Authors:** Hong Yu, Solomiya Nysak, Noemi Garg, Edison Ong, Xianwei Ye, Xiangyan Zhang, Yongqun He

**Affiliations:** 1 Department of Pulmonary and Critical Care Medicine, Guizhou Provincial People’s Hospital, Guiyang, 550002 Guizhou China; 2 Guizhou University Medical College, Guiyang, 550025 Guizhou China; 3 College of Literature, Science, and the Arts, University of Michigan, Ann Arbor, MI 48109 USA; 4 College of Pharmacy, University of Michigan, Ann Arbor, MI 48109 USA; 5 Department of Computational Medicine and Bioinformatics, University of Michigan Medical School, Ann Arbor, MI 48109 USA; 6 Unit for Laboratory Animal Medicine, Department of Microbiology and Immunology, Center for Computational Medicine and Bioinformatics, and Comprehensive Cancer Center, University of Michigan Medical School, Ann Arbor, MI 48109 USA

**Keywords:** Drug, Ontology, Drug adverse event, Ontology of drug adverse events, ODAE, Bioinformatics

## Abstract

**Background:**

Drug adverse events (AEs), or called adverse drug events (ADEs), are ranked one of the leading causes of mortality. The Ontology of Adverse Events (OAE) has been widely used for adverse event AE representation, standardization, and analysis. OAE-based ADE-specific ontologies, including ODNAE for drug-associated neuropathy-inducing AEs and OCVDAE for cardiovascular drug AEs, have also been developed and used. However, these ADE-specific ontologies do not consider the effects of other factors (e.g., age and drug-treated disease) on the outcomes of ADEs. With more ontological studies of ADEs, it is also critical to develop a general purpose ontology for representing ADEs for various types of drugs.

**Results:**

Our survey of FDA drug package insert documents and other resources for 224 neuropathy-inducing drugs discovered that many drugs (e.g., sirolimus and linezolid) cause different AEs given patients’ age or the diseases treated by the drugs. To logically represent the complex relations among drug, drug ingredient and mechanism of action, AE, age, disease, and other related factors, an ontology design pattern was developed and applied to generate a community-driven open-source Ontology of Drug Adverse Events (ODAE). The ODAE development follows the OBO Foundry ontology development principles (e.g., openness and collaboration). Built on a generalizable ODAE design pattern and extending the OAE and NDF-RT ontology, ODAE has represented various AEs associated with the over 200 neuropathy-inducing drugs given different age and disease conditions. ODAE is now deposited in the Ontobee for browsing and queries. As a demonstration of usage, a SPARQL query of the ODAE knowledge base was developed to identify all the drugs having the mechanisms of ion channel interactions, the diseases treated with the drugs, and AEs after the treatment in adult patients. AE-specific drug class effects were also explored using ODAE and SPARQL.

**Conclusion:**

ODAE provides a general representation of ADEs given different conditions and can be used for querying scientific questions. ODAE is also a robust knowledge base and platform for semantic and logic representation and study of ADEs of more drugs in the future.

**Electronic supplementary material:**

The online version of this article (10.1186/s12859-019-2729-1) contains supplementary material, which is available to authorized users.

## Background

As defined by the US FDA, an adverse drug event (ADE), also call adverse drug reaction is any untoward medical occurrence associated with the use of a drug in humans, whether or not considered drug related. ADEs are far more than one would think. ADEs are ranked as the fourth leading cause of death in the United States and Canada behind heart disease, cancer, and stroke [1]. In Europe, approximately 5% of all hospital admissions are caused by ADEs, and ADEs cause 197,000 deaths annually throughout the EU [2]. ADEs are estimated as the sixth leading cause of death worldwide. ADE-related death rates are associated with age, race, and urbanization subgroups [3]. However, how ADEs are associated with different factors is still unclear and not well studied.

Ontologies play critical roles in data science to facilitate data normalization, integration, processing, and analyses [4–7]. Biological/biomedical ontologies are sets of computer- and human-interpretable terms and relations that represent entities in the biological/biomedical world and how they relate to each other [8]. Ontologies have been widely used in various areas. For example, the NCBI Taxonomy ontology (NCBITaxon) includes the names and hierarchy of over one million of taxonomy names [9]. The Biological Pathway Exchange (BioPAX) standard ontology targets for representing molecular and cellular pathways and facilitates the exchange of biological pathway data [10]. The Gene Ontology (GO) [11] has been widely used to support gene expression data analyses, literature mining, and knowledge representation. Since its publication in 2000, the original GO paper [11] has been cited by over 20,000 publications. The Ontology for Biomedical Investigations (OBI) [12], co-developed by over 20 biomedical communities, provides integrative representations of data in various areas of life-science and clinical investigations [13–16]. Ontologies are also being used to support various metadata generations [15, 17, 18], leading to reproducible research, and data FAIRness (findable, accessible, interoperable, and reusable) [5, 19–21].

The Ontology of Adverse Events (OAE) is a community-based biomedical ontology in the area of adverse events [22, 23]. Under OAE, an adverse event is defined as a pathological bodily process that occurs after the administration of a pharmaceutical product. Such AE may result in any unfavorable and unintended sign (including an abnormal laboratory finding), symptom, or disease temporally associated with the use of a medicinal product. Aligned with the US FDA definition, an OAE-defined AE does not necessarily have a causal relationship with this treatment. OAE has been used in different applications, such as data integration for nanomaterial risk assessment [24], AE case report analysis [23], time information representation of AEs for temporal analysis [25], extraction of potential drug AEs from medical case reports [26], and drug class effect analysis [27]. OAE has also been extended to generate the Ontology of Vaccine Adverse Events (OVAE) [28], Ontology of Drug Neuropathy Adverse events (ODNAE) [29], and Ontology of Cardiovascular Drug AEs (OCVDAE) [27]. ODNAE focuses on representing drug-associated neuropathy AEs [29]. OCVDAE focuses on AEs associated with cardiovascular drugs used to treat cardiovascular diseases [27].

There is a critical need to develop a general purpose ontology of drug adverse events. ODNAE and OCVDAE focus on specific areas of ADE domains, cover overlapping drugs, and do not consider the effects of specific conditions such as patient age and drug-treated diseases on the outcomes of ADEs. Most ADE websites only list general ADEs and do not differentiate different AEs under specific age range such as adult, pediatric, and senior ages. Often a drug is used to treat different diseases. Given different diseases treated, the AEs induced by the drug may vary a lot. Although we were developers of the ODNAE and OCVDAE, we realize that it would be very beneficial to have a more general ADE ontology design to cover all kinds of scenarios. Towards this goal, here we report our design, implementation, and application of an open source and community-driven Ontology of Drug Adverse Events (ODAE). ODAE ontologically represents drugs, their chemical ingredients, AEs, patient age, drug treated diseases, and how these entities are related. Our use case is the collection of AEs associated over 200 neuropathy-inducing drugs through manual curation of related FDA package insert documents. We have also demonstrated the usages of ODAE by SPARQL querying for scientific questions and analyzing AE-specific drug class effects. Our studies show that a systematical analysis of the ODAE knowledge allows the identification of scientific insights about these drug AEs given specific age and disease conditions.

## Methods

### Collection of new AEs associated with FDA-approved neuropathy inducing drugs

While the ODNAE ontology [29] has stored the information of over 200 neuropathy-inducing drugs and their associated neuropathy AEs, ODNAE does not include other non-neuropathy AEs associated with these drugs. Our first effort was to survey FDA package insert documents and other related resources databases such as Drugs@FDA to identify all known AEs associated with these drugs. All data were compiled in an Excel file with a predefined format. Standard ontologies were used to represent related entities. Specifically, all AEs, diseases, and drugs were mapped to specific terms in OAE, the Disease Ontology (DOID) [30], and National Drug File Reference Terminology (NDF-RT) [31], respectively. Some adverse events were not associated with any OAE term, and were therefore compiled in a separate Excel file, which would be later manually annotated and used to update the OAE ontology.

### ODAE ontology generation

The ODAE ontology design follows the Open Biomedical Ontologies (OBO) Foundry principles (e.g., openness and collaboration) [32]. If ever possible, ODAE reuses terms from existing reliable ontologies, for example, NCBITaxon for organisms [9], UBERON for animal anatomical entities [33], the Disease Ontology (DOID) for diseases [30], and NRF-RT for drugs and drug-related information [31], and OAE for AEs [34]. For those AE terms not found in OAE, we added new OAE terms based on the standard OAE development procedure [34]. Ontofox (http://ontofox.hegroup.org/) [8] was used to extract subsets of related terms from different ontologies. Ontorat (http://ontorat.hegroup.org/) [35] was used to quickly add the annotations and relations between different entities. ODAE is formatted using the W3C standard Web Ontology Language (OWL2) (http://www.w3.org/TR/owl-guide/). The Protégé 5.0 OWL ontology editor (http://protege.stanford.edu/) was used for manual ontology editing.

### ODAE access, visualization, and licensing

The ODAE project website is located at GitHub: https://github.com/ODAE-ontology/ODAE. ODAE was also deposited in Ontobee, the default OBO Foundry ontology linked server [36]. The ODAE website in Ontobee is: http://www.ontobee.org/ontology/ODAE. The ODAE source code is freely available under the Creative Commons 3.0 License (http://creativecommons.org/licenses/by/3.0/), which allows ODAE users to freely distribute and use ODAE.

### ODAE ontology knowledge query and analysis

The ODAE ontology is formatted using the Web Ontology Language (OWL) [37] format. After ODAE is deposited to the Ontobee RDF triple store [36], SPARQL (a recursive acronym for SPARQL Protocol and RDF Query Language) [38] was used to query ODAE to address specific questions using the Ontobee SPARQL query web page (http://www.ontobee.org/sparql). The knowledge stored in the ODAE ontology was also analyzed using tools available under the Protégé OWL editor.

## Results

### Collection and analysis of various ADEs associated with 224 neuropathy-inducing drugs

In total 224 neuropathy-inducing drugs were annotated and analyzed in our study. In total 185 AEs were identified amongst these drugs. Only 14 AEs of the 185 AEs were not found in OAE, which were then annotated and added to OAE using standard OAE development protocol. Table 1 lists the top ten AEs associated with these drugs. Nausea is the most commonly identified ADE that occurs in 259 cases. Since each drug may be studied in over one scenario based on age and disease to be treated, the nausea AE cases is more than the total number (i.e., 224) of drugs studied. Other common adverse events include diarrhea, headache, vomiting, dizziness, rash, etc. (Table 1). The study also found that amongst the diseases and disorders treated or prevented by neuropathy inducing drugs are cancer, infections such as bacterial or fungal infections and immune disorders such as acquired immunodeficiency syndrome. Among the 224 neuropathy-inducing drugs we collected, over 30 drugs (e.g., sirolimus, linezolid, and levofloxacin) have been used to treat various respiratory diseases.

**Table 1:** Top ten ADEs associated with neuropathy-inducing drugs

Adverse Events	Frequency
Nausea	259
Diarrhea	173
Headache	168
Vomiting	149
Dizziness	106
Rash	99
Abdominal Pain	91
Fatigue	78
Infection	75
Constipation	66

### Collection and analysis of the ADE data given different conditions

**Fig. 1:**
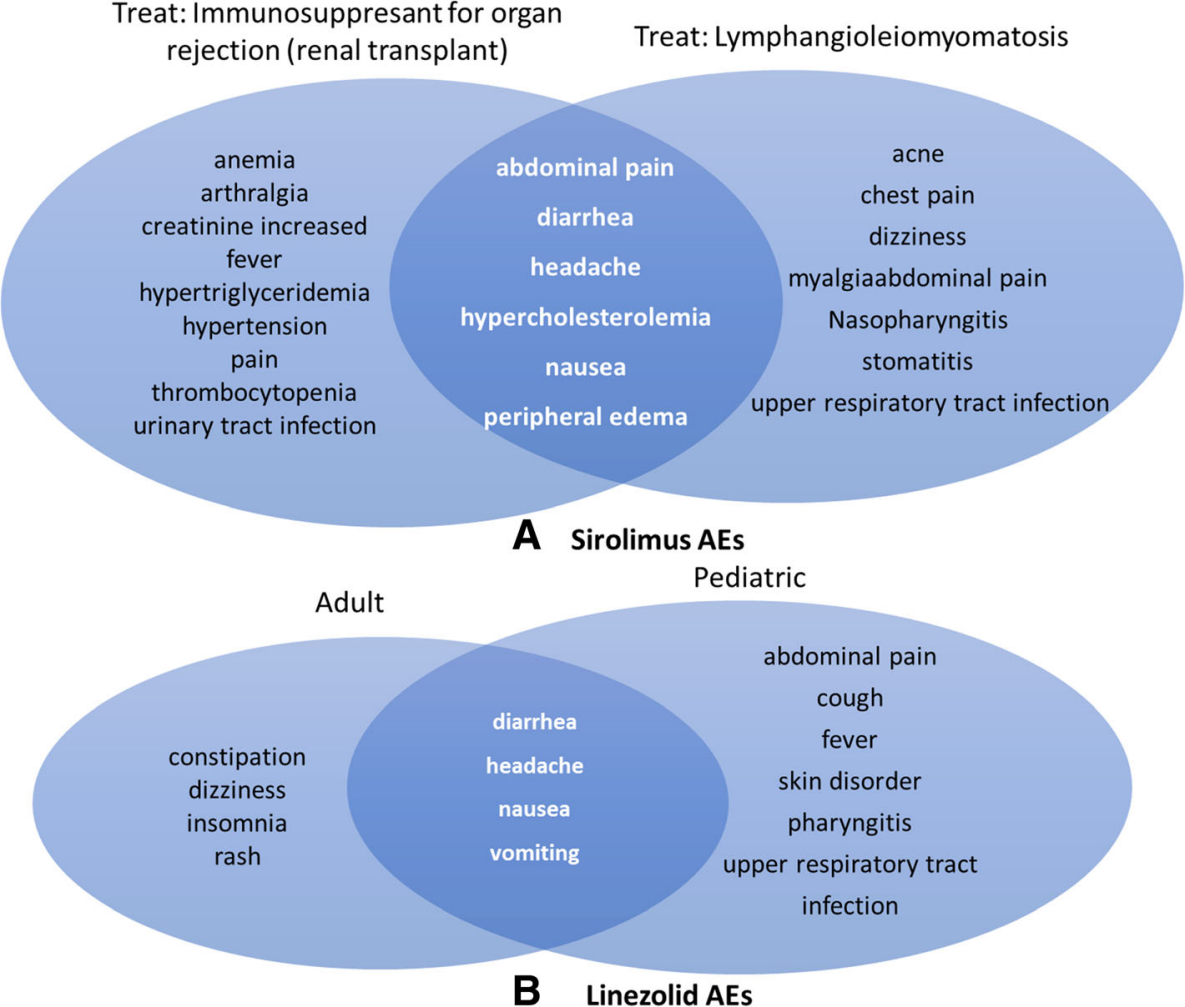
Use cases of drug use cases dependent on treated disease and age. **a** Sirolimus AEs found when Sirolimus was used to treat immunosuppressant for organ rejection in renal transplant. **b** Linezolid AEs when linezolid was used in adults or pediatric patients

Our study found that patient age and disease conditions have significant influence on the final ADE outcomes. For example, sirolimus, also known as rapamycin, is a macrolide compound used to prevent organ transplant rejection and to treat lymphangioleiomyomatosis [39, 40]. Sirolimus has immunosuppressant functions in humans and is commonly useful for preventing the rejection of kidney transplants. Sirolimus inhibits activation of T cells and B cells by reducing their sensitivity to interleukin-2 through mTOR inhibition. Sirolimus is also used to treat lymphangioleiomyomatosis, a rare, progressive lung disease that primarily affects women of childbearing age. The common sirolimus AEs associated with its usage for these two treatments include abdominal pain, diarrhea, headache, hypercholesterolemia, nausea, and peripheral edema (Fig. 1a). However, sirolimus also shows many AEs present only during its treatment of lymphangioleiomyomatosis or during its usage in renal transplantation (Fig. 1a). Linezolid is an antibiotic used to treat infections caused by aerobic Gram-positive bacteria (e.g., streptococci and vancomycin-resistant enterococci) [41]. Linezolid is often used for infections of the skin and pneumonia that are resistant to other antibiotics. Linezolid can be used in adults as well as pediatric patients. Our annotation of FDA package insert documents also found shared and differentiated AEs when linezolid is used in these two groups of patients (Fig. 1b).

Our study also found that the age and disease conditions have been found to be an important factor of the AEs of many other drugs, including Ribavirin, Pramipexole (mirapex), Nevirapine, recombinant Interferon-2a (Roferon A), Infliximab (remicade), Capecitabine (xeloda), and Alosetron (lotronex). As a drug example, Infliximab (remicade) is associated with different AEs given different ages (e.g., adults and pediatric), and disease conditions (i.e., Crohn’s disease, and ulcerative colitis). For instance, our manual annotation found that Infliximab is statistically significantly associated with neutropenia and leukopenia in Crohn’s disease patients but not in ulcerative colitis patients, and associated with pharyngitis and abdominal pain in ulcerative colitis patients but not in Crohn’s disease patients.

### ODAE design and development

**Fig. 2:**
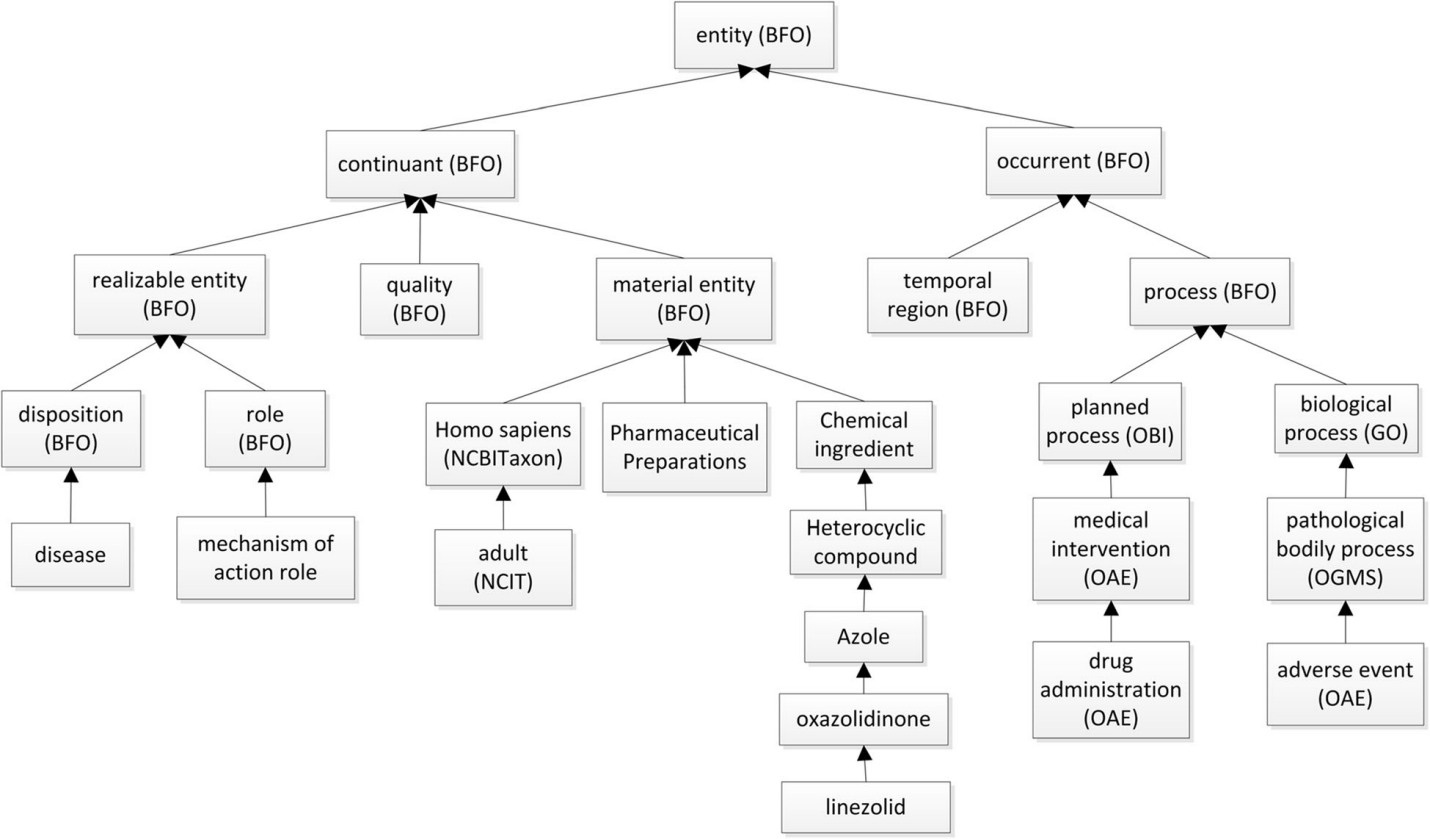
ODAE top level hierarchy with exampled specific terms. Each arrow represents an “is a” relation. Those terms with no ontology indicated are terms from NDF-RT

Figure 2 shows the basic top-level hierarchical structure of ODAE. Specifically, ODAE uses and is aligned with the Basic Formal Ontology (BFO) [42] upper level ontology. BFO has two branches: continuant and occurrent. The BFO continuant represents time-independent entities such as material entity (e.g., drug), quality and roles. The BFO occurrent represents time-related entities such as process (e.g., adverse event and drug administration) and time. BFO is adopted by over 100 biomedical ontologies (http://basic-formal-ontology.org/users.html). The usage of BFO makes ODAE easily and effectively integrated with other ontologies. ODAE also reuses terms from many ontologies. For example, ODAE reuses OAE to represent various AEs, the NCBI taxonomy ontology (NCBITaxon) [9] to represent human (i.e., *Homo sapiens*), and NDF-RT to represent drugs, drug ingredients, and mechanisms of action.

It is noted that the Disease Ontology (DOID) [43] and the Chemical Entities of Biological Interest (ChEBI) [44] have been recommended as the standard ontology to represent human diseases and chemicals, respectively. However, NDF-RT includes many diseases and the relations between drugs and diseases that may be treated by the drugs. Since such disease-drug information is not listed in DOID, we have also automatically extracted the disease-drug information from NDF-RT and use it in ODAE. Similarly, NDF-RT includes the terms of chemical ingredients as active components of drugs, which is not provided in ChEBI. Therefore, we also extracted the information of chemical ingredients from NDF-RT to ODAE. Our manual evaluation suggests that NDF-RT disease and chemical representations are also accurate and present the reality of the fields.

**Fig. 3:**
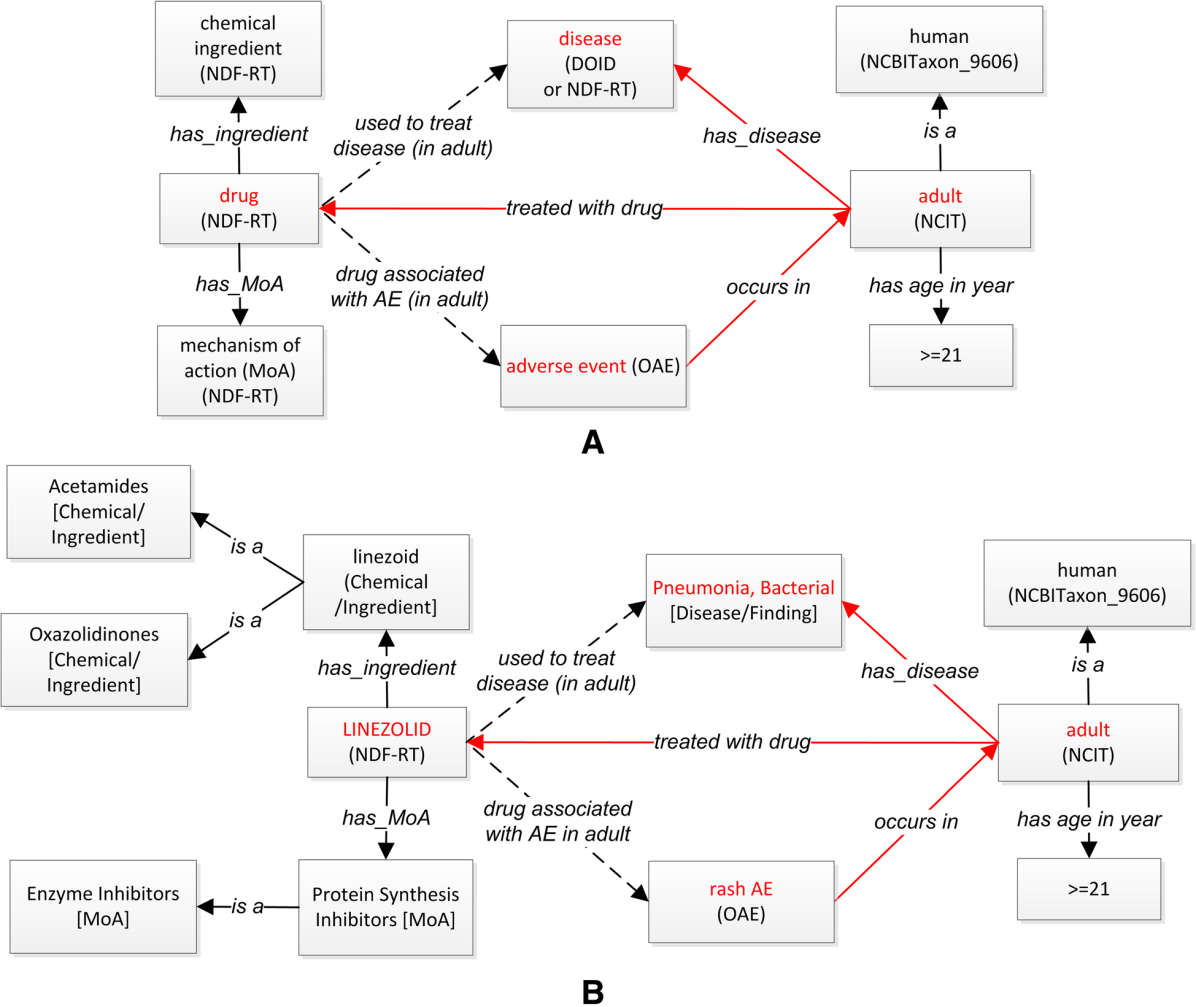
ODAE ontology design pattern and illustration. **a** General ODAE design pattern. **b** Illustration with the Linezolid drug inducing rash AE in adult. Note that the shortcut relation ‘used to treat disease (in adult)’ has the domain of drug and the range of disease, and the relation ‘drug associated with AE (in adult)’ has the domain of drug and the range of adverse event

Figure 3a provides a general design pattern that links drug, drug chemical ingredient, mechanism of action, human group (e.g. adult) based on age, disease treated by drug, and AE. In RDF and OWL, a property is a binary relation. In our ODAE, we need to represent complex relations, called *n-ary* relations, among more than two entities. To represent such relations, we have designed and applied different object properties as indicated in the arrows in Fig. 3. The object properties ‘used to treat disease (in adult)’ and ‘drug associated with AE (in adult)’ are the shortcut relations that semantically and directly link drug to disease and AE in patient (e.g., adult patient), respectively. As an example, Fig. 3b uses drug Linezolid to illustrate how the general design pattern works out. Based on the general design pattern (Fig. 3a), we can generate axioms for Linezolid as follows:

LINEZOLID: (‘drug associated with AE’ some (‘rash AE’ and (‘occurs in’ some Adult and (‘has disease’ some ‘Pneumonia, Bacterial’))))

The same information can also be presented using a simplified version for more efficient SPARQL query:

LINEZOLID: (‘drug associated with AE in adult’ some ‘rash AE’) and (‘used to treat disease’ some ‘Pneumonia, Bacterial’)

**Fig. 4:**
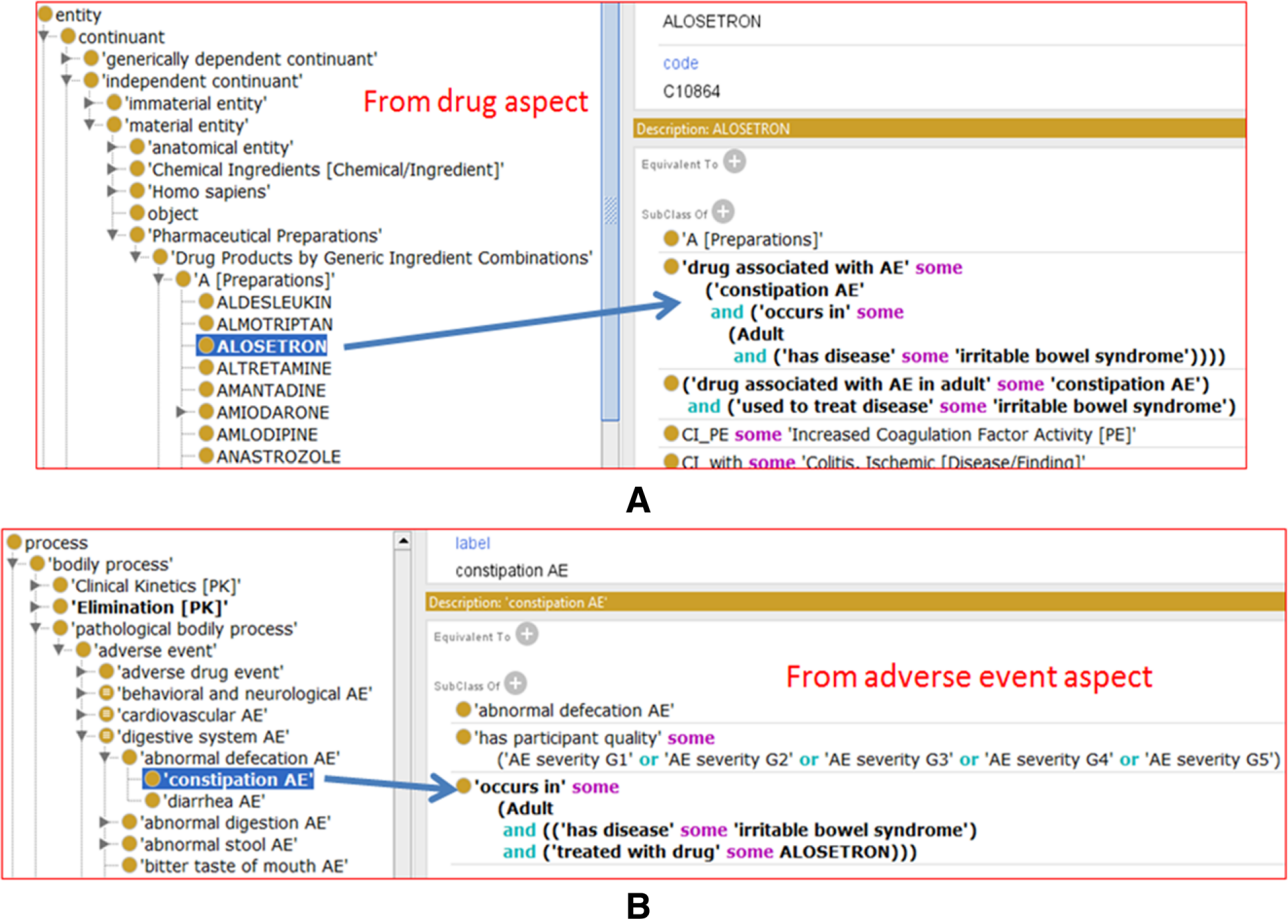
ODAE representation of Alosetron-associated constipation AE when the drug is used to treat adult irritable bowel syndrome. The representation can start from the drug side (**a**) or from the AE side (**b**). The relations among entities are represented based on the design pattern shown in Fig. 2. The terms are their properties are under the overall hierarchical structure shown in Fig. 3

Figure 4 further provides an ontology representation example using the drug Alosetron, which illustrates the ODAE hierarchy and the usage of design pattern (Fig. 3). Alosetron is a 5-HT3 antagonist used to treat diarrhea and abdominal discomfort that occurs in some women with irritable bowel syndrome [45]. As shown in Fig. 3, we can represent the same ADE information from the drug side or from the AE side. Figure 4a demonstrates how we can represent the ADE from the drug side, similar to the Linezolid example described above. To help users’ browsing and analysis from different angles, Fig. 4b illustrates the representation of the same ADE information from the AE side. From the ADE side, we can generate the following axiom:

‘constipation AE’: ‘occurs in’ some (Adult and ((‘has disease’ some ‘irritable bowel syndrome) and (‘treated with drug’ some ALOSETRON)))

Like ODNAE [29], ODAE represents the knowledge of drug adverse events from the FDA drug package insert documents. The detailed sample size information is typically available in package insert documents but not represented in ODAE. The knowledge represented in ODAE includes the statistically significant results out of the randomized controlled clinical trials described in the package insert documents. To provide the source of evidence and ensure the accuracy, ODAE also includes the drug package insert documents as reference annotations.

### ODAE statistics and access

As of January 12, 2019, ODAE has 3938 terms, including 3673 classes, 130 object properties, 18 data type properties, 114 annotation properties, and 3 instances. Most of the terms in ODAE are extracted and reused from over 20 ontologies (e.g., BFO, OAE, NCBITaxon, and DOID). ODAE only generates 15 new terms whose ontology identifiers having the “ODAE_” prefix. These 15 ODAE terms include 13 object properties, such as ‘drug AE occurs in’, ‘drug associated with AE’, and ‘treated with drug’. More ODAE statistics details can be found on the Ontobee website: http://www.ontobee.org/ontostat/ODAE.

It is noted that although ODAE does not have many new ODAE-specific terms, ODAE has generated many new semantical relations that link different drugs, AEs, human group with different age ranges (e.g., adult), and diseases to be treated by drugs (Figs. 3 and 4). In addition, ODAE semantically lists and annotates all these terms in an integrative hierarchical framework (Fig. 2).

ODAE is open-sourced and freely available. The ODAE ontology has been submitted to the Ontobee ontology repository and is openly available for visualization, browsing, and query on Ontobee: http://www.ontobee.org/ontology/ODAE. The following website provides one example of the Ontobee display of the drug ALOSTERON’s information in ODAE:

- http://www.ontobee.org/ontology/ODAE?iri=http://evs.nci.nih.gov/ftp1/NDF-RT/NDF-RT.owl%23N0000148640. The information shown in Fig. 3a can also be seen on this Ontobee page, and more information is provided as well.

- ODAE has also been deposited in the BioPortal website for web browsing and query: http://bioportal.bioontology.org/ontologies/ODAE.

### SPARQL query of the ODAE knowledge base

**Fig. 5:**
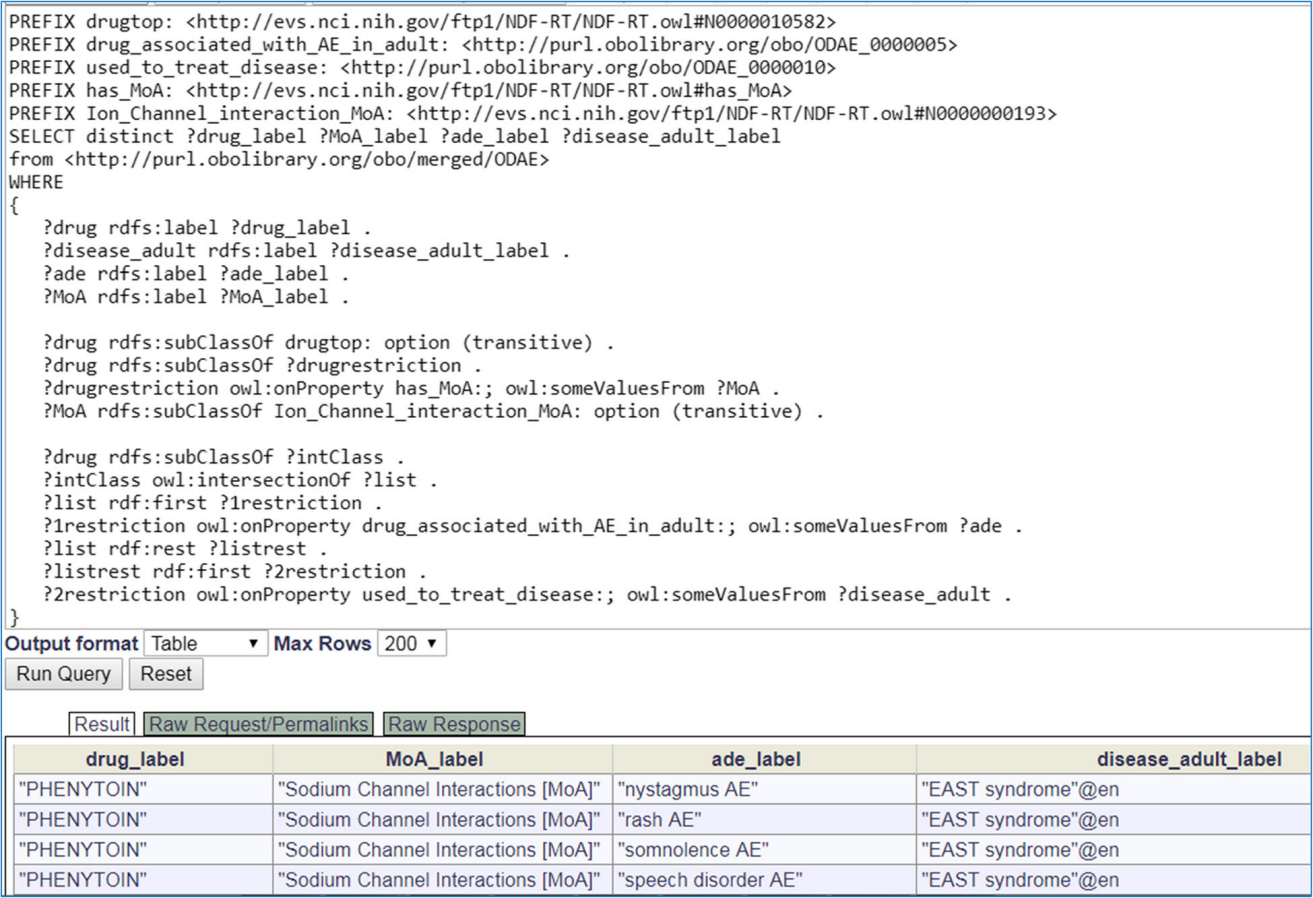
Example SPARQL query of ODAE. This query searches ODAE for all possible drugs that have some mechanism of action (MoA) related to ion channel interactions, and when used to treat some disease in adult patients, induce specific adverse events (AEs). Five related terms are defined using PREFIX. This query identified 8 drugs with 7 ion channel interaction MOAs, and these drugs are associated with 36 AEs when used to treat 8 diseases. Only part of results is shown in the screenshot. This query was performed using Ontobee SPARQL website (http://www.ontobee.org/sparql)

The ODAE knowledge and its semantic links to other ontologies can be efficiently queried using SPARQL. Figure 5 provides an Ontobee SPARQL query example, which queries all the drugs in ODAE that function by interacting with ion channels, drug-treated diseases, and AEs when the drugs are used to treat the diseases in adult patients. The short SPARQL identified 8 neuropathy-inducing drugs that have the mechanisms of actions (MoAs) of 7 different ion channel interactions, and 36 AEs are associated with these drugs when the drugs are used to treat 8 diseases in adults. The complete SPARQL results are available in the Additional File 1. One example is Phenytoin, an anti-seizure medication that functions by causing voltage-dependent block of voltage gated sodium channels [46]. EAST syndrome is a syndrome of seizures, sensorineural deafness, ataxia, mental retardation, and electrolyte imbalances [47]. When the drug is used to treat adult EAST syndrome patient, it can cause 7 AEs such as nystagmus, rash, somnolence, and speech disorder (Fig. 5). Another drug example is Diltiazem, a nondihydropyridine calcium channel blocker used in the treatment of hypertension, angina pectoris, and arrhythmia [48]. Our query found that Diltiazem is associated with asthenia AE when it is used to treat intermediate coronary syndrome in adults, and it is associated with 5 other AEs (i.e., pharyngitis, rhinitis, headache, constipation, and cough increased) when used to treat hypertension in adult patients. This example also shows that the same drug can induce different AEs when it is used to treat different diseases.

The above query example semantically links drugs, drug MoAs, AEs, and adult diseases treated by the drugs. Here the drugs and drug MoAs are recorded in NDF-RT, AEs represented in OAE, and diseases represented in DOID. Object properties are used to semantically link these terms to form specific knowledge information. Note that these drugs, MoAs, AEs, and diseases are organized in their own hierarchies. Based on the hierarchy, we can then query all possible ion channel interaction MOAs (e.g., sodium channel blocker) using the transitive query option (Fig. 5). Although not shown, more hierarchies (e.g., drug classification) can also be queried. Therefore, ODAE provides a rich semantic knowledge environment useful for flexible queries.

Furthermore, the SPARQL queried ODAE results can be used for statistical analysis. For example, we have recently generated a new ontology-based method to identify AE-specific drug class effect [27]. Such AE-specific drug class effect is defined to exist when all the drugs in a drug class (e.g., a specific MoA) are associated with an AE, which can be formulated as a proportional class level ratio (“PCR”) = 1. The PCR score is the ratio between the number of drugs in a class that are associated with an AE and the total number of drugs in the class [27]. Based on the PCR method [27], we identified many AE-specific drug class effects from ODAE. For example, we found that the tyrosine kinase inhibitor MoA class has a class effect with several AEs, including rash, fever, nausea, edema, dyspnea, and vomiting AEs. All the three drugs that function with their MoAs under the MoA class, including IMATINIB (has_MoA Bcr-Abl Tyrosine Kinase Inhibitors [MoA]), ERLOTINIB (has_MoA HER1 Antagonists [MoA]), and TRASTUZUMAB (has_MoA HER2/Neu/cerbB2 Antagonists [MoA]), are associated with these AEs in drug-treated adult patients. We also found that 9 out of 10 drugs with the Immunologic and Biological Factors [MoA] are associated with the nausea AEs, leading to a PCR of 0.9 between the MoA class and the nausea AE. Such classification of AE-specific drug class effects rely on the semantic relations and hierarchical definitions in ODAE, demonstrating another usage of the ODAE knowledge in identifying possible causal associations.

## Discussion

The contribution of this manuscript is multifold. First of all, we found and used an ontology-based approach to annotate all cases of neuropathy-inducing drug AEs given patient age and diseases, which are reported in the formal FDA-released drug package insert documents. Our study found that given different patient age and diseases, drugs tend to generate overlapping but different profiles of adverse events. Such a phenomenon may have important implication for how to report ADEs in public media (e.g., web reports). Second, we developed an Ontology of Drug Adverse Events (ODAE). ODAE includes an interoperable and integrative hierarchical structure and an ontology design pattern that logically links different types of entities, which were then applied to represent all aforementioned ADEs including those ADEs conditional to the changes of patient age and diseases to be treated. Third, our use case testing and ODAE evaluation found that ODAE can serve as a knowledge base of ADEs and be used for computer-assisted advanced queries. Since our ODAE approach is generic and scalable, it is also possible to apply the ODAE platform to represent other types of ADEs.

ODAE overlaps with but significantly differs from the ODNAE ontology. The Ontology of Drug Neuropathy Adverse events (ODNAE) focuses on ontological representation of drug-associated neuropathy AEs [29]. At current stage, ODAE and ODNAE include similar number of drugs. However, the ADEs represented in ODNAE are only neuropathy AE or its subclasses. Since neuropathy-inducing drugs also induce non-neuropathy AEs, ODNAE has its shortcoming in systematic representation of all possible AEs significantly associated with these over 200 drugs. ODAE first solved this issue. Since our recent analysis identified many ADEs that occur in a way dependent on patient age and diseases, ODAE has a new goal to logically and efficiently representing ADEs given patient age and disease, which is not done in ODNAE. To be more general and robust, our new ODAE design pattern is much simpler than the ODNAE pattern. For example, ODNAE includes many intermediate terms like ‘bupropion-associated neuropathy AE’ (ODNAE_0000043). Using such logic, to deal with age and drug-treated disease-specific ADEs, ODAE would need to generate many new terms such as ‘bupropion-associated neuropathy AE in adult (or other age range)’ and ‘bupropion-associated neuropathy AE when treating disease X’ However, such an approach would result in the generation of too many new terms in ODAE and is not scalable. Therefore, we decided not to include all these intermediate layer terms. Indeed, these intermediate terms were not used in any of the figures shown in the ODNAE paper [29]. Our evaluation found that the exclusion of these intermediate terms does not change the performance of robust queries of ODAE. Furthermore, while ODNAE focuses on neuropathy-inducing drugs, ODAE will become a general platform for representing AEs associated with other drugs in different domains.

Similarly, ODAE also differs from the Ontology of Cardiovascular Drug AEs (OCVDAE) [27]. OCVDAE targets on cardiovascular drugs used to treat cardiovascular diseases [27]. ODAE is more general. The design pattern in ODAE is also more robust than OCVDAE. One specific demonstration of OCVDAE is its application in analyzing drug class effect, which is defined exist when all the drugs in a drug class are associated with an AE. In the OCDAE paper, a mathematic proportional class level ratio (PCR) formula was developed and used to identify different types of drug class effect, such as the finding of cardiovascular drugs under the active transporter interactions class (including reserpine, indapamide, digoxin, and deslanoside) has statistically significant drug class effect on anorexia and diarrhea AEs [27]. At least theoretically, ODAE can also be used to analyze such drug class effects since ODAE also includes NDF-RT drug classifications and drug AEs. We will perform further studies to evaluate and possibly extent this potential.

ODAE is anticipated to play an important role in the future semantic ADE representation and drug safety studies. The Ontology of Adverse Events (OAE), developed by our group and collaborators around the world, has been a major contribution in recent years that lays out the semantic framework and representation of various adverse events. The OAE general framework has been applied to represent and study AEs associated with vaccines in the Ontology of Vaccine Adverse Events (OVAE) [28], neuropathy-inducing drugs in ODNAE [29], and drugs used to treat cardiovascular diseases in OCVDAE [27]. OAE also provides a way to link different domains of entities such as medical administration, anatomic entities, phenotypes, and patient qualities, and cross-reference other controlled terminologies such as the Common Terminology Criteria for Adverse Events (CTCAE) [49] and the Medical Dictionary for Regulatory Activities (MedDRA) [50]. It has become clear that biological ontologies support the integration of biological knowledge for learning and predicting ADEs [51]. ODAE provides a new and integrative ontology towards systematical representation of all ADEs and how these ADEs are related to drugs, drug mechanisms of action, drug chemical ingredients, etc. ODAE can thus serve as an ADE knowledge base and a hub to link other related fields of knowledge and studies.

A future direction is how to further study and represent the molecular mechanisms of the adverse drug events. Our Fig. 5 SPARQL query example demonstrates how we can query ODAE for the semantic relations among drugs, drug MoAs, and diseases treated by the drugs, and AEs that occurs in adults after treatment with the drugs. The ontology hierarchy provides us a feasible strategy to examine the effect of a specific group of entities (e.g., the group of ion channel interaction MoAs). Similarly, we can also search for information about other related entities such as drug ingredients and their hierarchies. It is noted that the mechanisms of why age and existing disease affect the ADEs are often unclear and require extensive studies. Humans with different age stages have many different physiological characteristics, habits, and living environments. The existence of diseases in patients may also change the preference and status of immune systems and metabolisms, which may also affect the body reactions to the administration of a drug. More knowledge obtained from experimental studies can be added to ODAE in the future. We have also shown in the Results section how to analyze AE-specific drug class effects [27] using ODAE, which can suggest causal association between the AE and the drug classification (e.g., MoA). Therefore, ODAE provides a knowledge base and platform to represent, integrate, and analyze different drugs and drug ingredients, diseases, AEs, mechanisms, and their intricate relations among these entities.

## Conclusion

In this study, we first collected different AEs associated with 224 neuropathy-inducing drugs by manual annotation of FDA-released drug package insert documents. Our study identified many ADEs conditional to the patient age and diseases to be treated with drugs. We then developed an ontological design pattern for representation of the relations between drugs, ages, diseases to be treated, and AEs, and based on develop the Ontology of Drug Adverse Events (ODAE). ODAE reuses existing ontologies and logically represents all the 224 drugs, drug chemical ingredients and their features, and their associated AEs. ODAE can be used for visualization and queries of ADEs. Overall, ODAE is a novel ontology and can serve as a new platform for representing the AEs and related information for other drugs.

## Additional file

Additional file 1: Complete results of SPARQL query in Fig. 5. (XLSX 17 kb)

## Abbreviations

AE: Adverse event
BFO: Basic Formal Ontology
FDA: Food and Drug Administration
NCBITaxon: NCBI taxonomy ontology
OAE: Ontology of Adverse Events
OBO: Open Biological/Biomedical Ontologies
OCVDAE: Ontology of Cardiovascular Drug AEs
ODAE: Ontology of Drug Adverse Events
ODNAE: Ontology of Drug Neuropathy Adverse events
OVAE: Ontology of Vaccine Adverse Events
OWL2: Web Ontology Language
RDF: Resource Description Framework
SPARQL: SPARQL Protocol and RDF Query Language
